# Efficacy and safety of lecanemab monoclonal antibody in the treatment of mild to moderate Alzheimer’s disease: a systematic review and meta-analysis

**DOI:** 10.3389/fphar.2026.1947129

**Published:** 2026-09-28

**Authors:** Shang-Ying Li, Jia-Xin Zhang, Long-Zhuan Huang, Han-Biao Wu, Yu-Bing Ye, Aiqun Liu, Jisheng Chen, Sha Lai

**Affiliations:** 1 Key Specialty of Clinical Pharmacy, The First Affiliated Hospital of Guangdong Pharmaceutical University, Guangzhou, China; 2 Department of Neurology, Neurological Research Institute of Integrated Traditional Chinese and Western Medicine, The First Affiliated Hospital of Guangdong Pharmaceutical University, Guangzhou, China

**Keywords:** Alzheimer’s disease, ARIA-E, ARIA-H, efficacy, lecanemab, safety

## Abstract

**Background:**

Lecanemab is a novel therapy for Alzheimer’s disease (AD), but comprehensive evidence is lacking. This systematic review and meta-analysis evaluated the efficacy and safety in AD.

**Methods:**

Registered in PROSPERO (CRD420251146867). PubMed, Embase, Cochrane Library, Web of Science, and Scopus were searched for English-language RCTs. Efficacy and safety were evaluated using the risk difference with a Mantel-Haenszel fixed-effects model. Study quality and sensitivity were assessed using Cochrane risk-of-bias and leave-one-out methods. Primary outcomes: mean changes in Alzheimer’s Disease Composite Score (ADCOMS), Clinical Dementia Rating Scale Sum of Boxes (CDR-SB), Alzheimer’s Disease Assessment Scale-Cognitive Subscale (ADAS-Cog), and amyloid burden on positron emission tomography (PET). Secondary: adverse events including amyloid-related imaging abnormalities with edema (ARIA-E), ARIA with hemorrhage (ARIA-H), death, treatment-emergent adverse events (TEAEs), headache, dizziness, and urinary tract infection.

**Results:**

A total of five articles involving 8 clinical trials were included. Based on the fixed-effect model, lecanemab treatment significantly improved cognitive performance on ADCOMS (SMD -0.1999, 95% CI [-0.2831, −0.1167], I^2^ = 0%, *P* < 0.00001), CDR-SB (SMD −0.1853, 95% CI [−0.2683, −0.1024], I^2^ = 0%, *P* < 0.0001), ADAS-Cog (SMD −0.1821, 95% CI [−0.2670, −0.0973], I^2^ = 0%, *P* < 0.0001) and amyloid burden on PET (SMD −1.1872, 95% CI [−2.2474, −0.1270], I^2^ = 96%, *P* = 0.03). Sensitivity analysis indicated stability. However, lecanemab was associated with an increased risk of TEAEs (MH RR 1.0730, 95% Cl [1.0296, 1.1183], I^2^ = 0%, *P* = 0.0008) and an increased risk of headache (MH RR 1.3617, 95% Cl [1.0243, 1.8103], I^2^ = 0%, *P* = 0.03). Sensitivity analysis indicated that both results were stable. The lecanemab group showed no significant differences in the risks of death, dizziness, or urinary tract infection compared with the control group. In addition, the lecanemab group showed a significantly increased risk of ARIA-E (MH RR 5.9948, 95% CI [3.9047, 9.2037], I^2^ = 86%, *P* < 0.00001) and ARIA-H (MH RR 1.7490, 95% CI [1.3896, 2.2014], I^2^ = 41%, *P* < 0.00001). Sensitivity analyses confirmed the stability of the TEAE finding, while the headache result should be interpreted with caution due to the limited number of studies. After excluding Honig et al. (OLE) 2021 via leave-one-out, results became stable, likely due to its open-label design.

**Conclusion:**

Lecanemab slowed cognitive decline versus placebo in mild-to-moderate AD by ADCOMS, CDR-SB, and ADAS-Cog. High heterogeneity in PET amyloid burden warrants future RCTs. Nevertheless, lecanemab was associated with an increased risk of TEAEs and headache.

**Systematic Review Registration:**

https://www.crd.york.ac.uk/PROSPERO/view/CRD420251146867.

## 1 Introduction

Alzheimer’s disease (AD) is a complex and progressive neurodegenerative disorder and represents the most common form of dementia worldwide. Its clinical manifestations are heterogeneous and multifaceted. In the early stage, patients typically present with impaired recent memory and declining executive function, followed progressively by deficits in visuospatial, language, executive, behavioral, or motor functions (Graff-Radford et al., 2021). In the advanced stage, patients eventually lose the ability to perform self-care entirely and frequently die as a result of related complications. AD accounts for approximately 60%–80% of the nearly 57 million dementia cases worldwide. Its prevalence is increasing at an accelerating rate, with the age-standardized prevalence projected to reach 821.80 per 100,000 population by 2040 (Hao and Chen, 2025). Moreover, it has become the sixth leading cause of death in the United States (Zheng and Wang, 2025). Because the percentage increase in life expectancy is greater in low-income countries than in high-income countries, the proportion of individuals living with dementia is expected to rise over time, posing substantial challenges to healthcare systems and socioeconomic development (Livingston et al., 2024).

For decades, the treatment of AD has relied primarily on symptomatic therapies, such as cholinesterase inhibitors (e.g., donepezil) and N-methyl-D-aspartate (NMDA) receptor antagonists (e.g., memantine) (Arvanitakis et al., 2019; Vaz and Silvestre, 2020). Although these agents can alleviate symptoms to some extent, they are unable to fundamentally slow disease progression or reverse its underlying pathological processes (Moss, 2020). Therefore, the development of innovative therapies capable of effectively halting or reversing disease progression has become an urgent priority. In recent years, monoclonal antibody therapies targeting beta-amyloid (Aβ), a key pathological hallmark, have brought a historic breakthrough to the treatment of AD. According to the amyloid cascade hypothesis, the abnormal deposition and aggregation of beta-amyloid (Aβ) constitute a central event in the pathogenesis of AD. Monoclonal antibodies targeting this pathway are designed to modify disease progression by promoting the clearance of cerebral Aβ plaques (Wu et al., 2022; Chowdhury, 2023). Early drug development efforts, such as solanezumab, which targets Aβ monomers, and aducanumab, which targets Aβ oligomers and fibrils, demonstrated a certain capacity to reduce Aβ plaque burden. However, their clinical trials failed to show significant cognitive benefits (Doody et al., 2014; Doggrell, 2018; Budd et al., 2022). In particular, aducanumab received controversial accelerated approval from the U.S. Food and Drug Administration (FDA), which ultimately led to its withdrawal from the market (Whitehouse and Saini, 2022). Despite these challenges, the development of anti-Aβ monoclonal antibodies has continued. Donanemab, which targets a pyroglutamate-modified form of Aβ (N3PG-Aβ) (Mintun et al., 2021), has shown positive results in phase III trials, significantly slowing cognitive decline in patients with early symptomatic AD (Sims et al., 2023), and received FDA approval in 2024 for the treatment of early symptomatic Alzheimer’s disease (Harris, 2024). These developments suggest that antibody strategies targeting distinct Aβ epitopes are gradually moving toward clinical validation.

Lecanemab is a humanized monoclonal antibody directed against soluble amyloid-β (Aβ) aggregates. Among the various forms of Aβ implicated in AD, lecanemab selectively binds to highly neurotoxic Aβ protofibrils and is believed to reduce the levels of Aβ protofibrils and amyloid plaques (Aβ plaques) in the brain (Niidome et al., 2024). Compared with other monoclonal antibodies (mAbs), lecanemab is distinguished by its preferential binding to smaller Aβ aggregates, a higher number of antibodies binding per aggregate, and its high affinity for these aggregates (Fertan et al., 2025). In addition, lecanemab not only effectively inhibits further Aβ aggregation but also promotes microglia-mediated clearance of pre-existing Aβ plaques. In the phase III clinical trial (CLARITY-AD), lecanemab slowed cognitive decline by 27% in patients with early AD after 18 months of treatment and significantly reduced cerebral Aβ deposition (van D et al., 2023). These milestone findings led to the full approval of lecanemab by the FDA in July 2023, making it the first monoclonal antibody fully approved for the treatment of AD and bringing new hope to the field of AD therapeutics (Lecanemab, 2023). However, despite its notable efficacy, safety concerns associated with lecanemab, particularly amyloid-related imaging abnormalities (ARIA), remain a major consideration in clinical practice. ARIA includes cerebral edema (ARIA-E) and cerebral microhemorrhages together with hemosiderin deposition (ARIA-H). These adverse events are believed to be associated with vascular inflammatory responses during Aβ clearance (van D et al., 2023). In addition, other common adverse reactions, such as headache, dizziness, and urinary tract infections, as well as rare reports of death, also warrant careful attention. These safety concerns impose stringent requirements on clinical practice, including regular magnetic resonance imaging (MRI) monitoring during treatment and strict patient screening and risk assessment prior to and during therapy.

Given the complexity of lecanemab in terms of both efficacy and safety, a systematic summary and meta-analysis of existing clinical trials is of great significance. We conducted an updated systematic review combined with meta-regression analysis to comprehensively evaluate the efficacy and safety of lecanemab in the treatment of AD. This study aims to provide clinicians with more direct and clinically useful insights into these emerging therapies, thereby guiding patient monitoring and offering references for future drug development.

## 2 Methods

### 2.1 Study types and search strategy

This study was conducted in accordance with the preferred Reporting Items for Systematic Reviews and Meta-Analyses (PRISMA) statement (Page et al., 2021). We comprehensively searched five databases, including PubMed, Embase, Cochrane Library, Web of Science, and Scopus, for full-text English publications from database inception to 10 July 2025.

The retrieval strategy was designed and implemented by experienced medical librarians based on researchers’ inputs. Relevant studies were identified by combining two search themes: the first was lecanemab, and the second was AD. Taking PubMed as an example, the full search strategy was as follows: ((“Alzheimer Disease” [Mesh] OR “Alzheimer Disease” OR “Alzheimer Syndrome” OR “Alzheimer-Type Dementia” OR “ATD” OR “Alzheimer’s Disease” OR “Alzheimer Dementia” OR “Senile Dementia” OR “Alzheimer Type Dementia” OR “Primary Senile Degenerative Dementia” OR “Alzheimer Sclerosis” OR “Sclerosis, Alzheimer” OR “Presenile Dementia” OR “Acute Confusional Senile Dementia” OR “Familial Alzheimer Disease” OR “FAD”) AND (lecanemab OR leqembi OR BAN2401 OR BAN 2401 OR BAN-2401)). The same strategy was adapted for the other databases using their respective syntax. In terms of inclusion criteria, all clinical studies on AD patients treated with lecanemab at a dose of 10 mg/kg once every 2 weeks were included, wich published in full-text English. There were no restrictions on age, gender, or study duration. In case of duplicate publications, only the one with the latest and most comprehensive information was included. We excluded studies with insufficient relevant data (e.g., ongoing studies with unpublished data), conference abstracts, case reports, editorials, reviews, and non-human studies. Three researchers (LSY, ZJX and HLZ) independently assessed study eligibility, and any discrepancies were resolved by another researcher (LS). Studies without a valid placebo-controlled group were also excluded.

### 2.2 Data extraction and outcome assessment

All data were independently extracted by three researchers (LSY, ZJX and HLZ) using a data extraction form, with study eligibility assessed simultaneously. Any discrepancies were resolved by another researcher (LS). Collected data included study characteristics such as first author, publication year, sample size, NCT number, diagnostic criteria for the disease, study duration, and mean changes from baseline to post-treatment in ADCOMS, CDR-SB, ADAS-Cog and amyloid burden on PET, as well as the incidence of ARIA-E, ARIA-H, death, TEAEs, headache, dizziness, and urinary tract infection, together with patients’ demographic and clinical characteristics.

For efficacy outcomes, only data from randomized controlled trials (RCTs) were included, consistent with the pre-specified PICOS framework. For safety outcomes, particularly ARIA, we also included data from the open-label extension (OLE) of the McDade et al. (McDade et al., 2022) study and Honig et al. (Honig et al., 2023) for supportive analysis. This approach was based on the methodological precedent established in a previously published systematic review and meta-analysis of lecanemab Arroyo-Pacheco et al. (Arroyo-Pacheco et al., 2025), which included OLE data alongside RCT data to maximize the available safety information, given the relative rarity of ARIA events and the extended follow-up provided by OLE designs. In the original phase 2 study by McDade et al. (McDade et al., 2022), the OLE phase was explicitly designed as a complementary extension of the randomized CORE phase to evaluate long-term safety and durability of treatment effects, and the authors reported that OLE data provided long-term safety information that could not be captured within the randomized period alone. Therefore, in the present analysis, OLE data were included only for safety outcomes (ARIA-E and ARIA-H) and were clearly distinguished from RCT data in all tables and figures. To minimize potential bias, sensitivity analyses were conducted using both fixed-effect and random-effect models, and the impact of including OLE data on the overall results was assessed by comparing analyses with and without the OLE data. OLE-derived findings were interpreted with caution, with appropriate caveats noted in the Discussion section.

Three published studies contained data from multiple RCTs across different trial phases. For instance, the lecanemab study reported by Logovinsky et al. included the multiple ascending dose (MAD) phase and the single ascending dose (SAD) phase (Logovinsky et al., 2016). In the present analysis, these phase comparisons were treated as independent RCTs in the primary analysis. This decision was based on the original study design: each phase had its own randomized, double-blind, placebo-controlled protocol, independent randomization, and distinct participant cohorts, and the results were reported separately in the original publication. To assess the potential impact of this approach on the overall findings, we conducted sensitivity analyses excluding both phases from the Logovinsky et al. study (Logovinsky et al., 2016). The Cochrane Risk of Bias Tool was adopted to assess the risk of bias of included RCTs (Hongqiu et al., 2014).

### 2.3 PICOS framework

The selection criteria for this systematic review and meta-analysis were defined according to the PICOS framework:

Population (P): Patients diagnosed with mild to moderate Alzheimer’s disease. The included RCTs enrolled patients with mild-to-moderate AD, as well as those with mild cognitive impairment due to AD (MCI due to AD) or mild AD dementia; the latter population aligns with the approved indication for lecanemab.

Intervention (I): Lecanemab monoclonal antibody. In terms of inclusion criteria, all clinical studies on AD patients treated with lecanemab at a dose of 10 mg/kg once every 2 weeks were included, which were published in full-text English. Treatment duration varied across studies, with the primary efficacy assessment conducted at 18 months.

Comparator (C): Placebo.

Outcomes (O): The primary efficacy outcome was the change in cognitive function, measured by the ADAS-Cog, and the change in functional ability, measured by the CDR-SB, both assessed at 18 months. Secondary outcomes included safety profiles, such as the incidence of adverse events, with particular attention to ARIA, including ARIA-E and ARIA-H, as well as TEAEs, headache, dizziness, and urinary tract infection.

Study design (S): We included RCTs of any phase (II-IV) that evaluated lecanemab in patients with AD. For safety outcomes, particularly ARIA and adverse events, we also included OLE studies of the eligible RCTs to maximize the detection of rare events and to capture extended safety follow-up data. Single-arm studies without a concurrent control group, observational studies, case reports, case series, reviews, and conference abstracts were excluded.

### 2.4 Outcome indicators

Primary outcomes included the mean changes from baseline in most cognitive measures reported in the original studies: ADCOMS, CDR-SB, ADAS-Cog, and Amyloid burden on PET. Secondary outcomes were events of ARIA-E, ARIA-H, death, TEAEs, headache, dizziness, and urinary tract infection. Total ARIA-E events were defined as any reported ARIA-E event of any severity, ranging from mild abnormalities detected on brain imaging (not always causing severe clinical symptoms) to more severe ARIA-E (resulting in serious consequences). ARIA-H included events reported as ARIA-H and microhemorrhages identified on MRI images as part of the predetermined procedures. Data were analyzed according to the intention-to-treat principle. According to the individual definitions provided in each study, TEAEs were generally classified as mild or moderate and included headache, dizziness, urinary tract infection, and infusion reactions, among others.

### 2.5 Data synthesis and analysis

The calculations and figures were performed using RevMan 5.4 or Stata Statistical Software version 16.0. All statistical tests used a two-sided α value of 0.05 as the criterion for statistical significance. In this study, a fixed-effects model was used for the meta-analysis of continuous and dichotomous variables reported for the experimental group (lecanemab treatment) and the control group in patients with AD, using SMD and risk ratio (RR) with 95% confidence intervals for comparison. In addition, different meta-analysis methods and the leave-one-out method were used for sensitivity analyses to further examine the validity and stability of the effect estimates. Heterogeneity levels were assessed using the I^2^ statistic, with an I^2^ value >50% considered indicative of statistically significant heterogeneity (Wang et al., 2023). Funnel plots were developed using RevMan version 5.4.

## 3 Results

### 3.1 Research selection and characteristics

The literature search identified 7,465 studies related to lecanemab, of which 2,854 were removed due to duplication, and 4,552 were excluded after initial screening by reading titles and abstracts. Additionally, 54 studies were further excluded, including 15 with no appropriate data, 6 duplicates of other studies, 9 not meeting population criteria, 7 conference abstracts and case reports, and 17 reviews and protocols. Finally, five full-text articles met all the inclusion criteria, comprising 8 clinical trials in which all patients had mild-to-moderate AD. A total of 3,469 patients received lecanemab as the experimental group, and 2,099 patients received placebo as the control group (van D et al., 2023; McDade et al., 2022; Honig et al., 2023; Logovinsky et al., 2016; Swanson et al., 2021). The search flow diagram is shown in Figure 1, and the detailed search strategy is presented in Supplementary Table S1.

**FIGURE 1:**
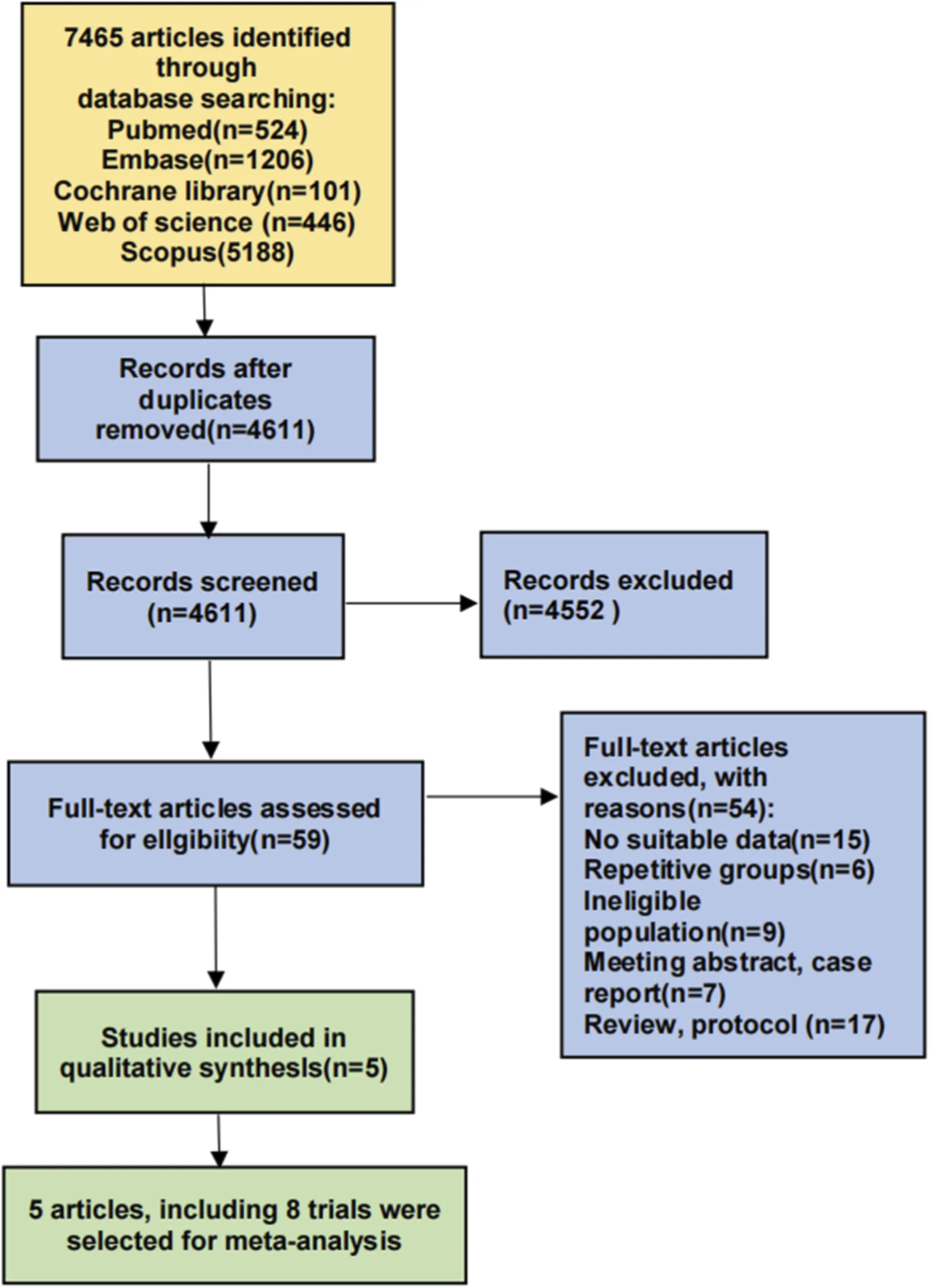
Flow chart of the assessment of the studies identified in the meta-analysis.

Regarding efficacy, 4 clinical trials reported the mean change from baseline for ADCOMS and CDR-SB, while 3 clinical trials reported the mean change from baseline for ADAS-Cog and Amyloid burden on PET, respectively. Regarding safety, a total of five clinical trials reported ARIA, including ARIA-E and ARIA-H, of which 4 were RCTs and 1 was an open-label extension (OLE) study. In addition, 4 RCTs reported TEAEs. Headache, dizziness, urinary tract infection, and death were each reported in 2 RCTs. The study population included individuals in the early stages of the AD continuum, covering patients with mild cognitive impairment (MCI) due to AD and mild AD dementia, with study durations ranging from 12 to 24 months. The characteristics and results of the included studies are summarized in Table 1. Supplementary Figure S1 also summarizes the risk of bias of the trials.

**TABLE 1:** Characteristics of clinical trials involving lecanemab.

References	Diseases	Diagnosis method	NCT	Study duration (months)	Age	Sex	Total number of patients (n)	
					Trial/Placebo Mean (SD)	Trial/Placebo (male %)	Trial	Placebo
Logovinsky et al. (MAD) 2016	Mild to moderate AD	National institute of neurological and communicative disorders and stroke and the Alzheimer’s disease and related disorders association; MMSE scores of 16–28	NCT01230853	12	70.0(9.97)/70.0(11.70)	45.8/75.0	24	8
Logovinsky et al. (SAD) 2016			NCT01230853	12	70.9(10.0)/72.1(9.2)	50.0/58.3	36	12
Swanson et al., 2021	Mild cognitive impairment due to AD or mild AD dementia	All subjects were confirmed amyloid positive via amyloid positron emission tomography (PET) or cerebrospinal fluid (CSF) Aβ1–42 for eligibility	NCT01767311	18	74.0(7.7)/71.1(8.9)	53.7/42.4	587	238
McDade et al., 2022		Meet NIA-AA core clinical criteria for MCI due to AD and probable AD dementia	NCT01767311	18	74.0(7.7)/71.1(8.9)	53.7/42.4	587	238
McDade et al., 2022			NCT01767311	18	74.0(7.7)/71.1(8.9)	53.7/42.4	587	238
Dyck et al., 2023		Meet NIA-AA core clinical criteria for MCI due to AD and probable AD dementia; amyloid positivity was determined by PET or CSF measurement of Aβ	NCT03887455	18	71.4 (7.9)/(71.0 + 7.8)	48.4/47.0	859	875
Honig et al. (CORE) 2023		/	NCT01767311	18	74.0(7.7)/71.1(8.9)	53.4/43.7	609	245
Honig et al. (OLE)2023		/	NCT01767311	24	74.0(7.7)/71.1(8.9)	51.7/43.7	180	245

For studies by the same author, the corresponding trial phase is indicated in parentheses to clearly distinguish between different trials. McDade et al., 2022 refers to the CORE, phase of the trial, and McDade et al., 2022 refers to the OLE, phase of the trial.

### 3.2 Meta-analysis results of the efficacy (primary outcomes) of lecanemab in patients with mild to moderate AD

Research indicates that, according to the meta-analysis, compared with the control group, treatment with lecanemab at 10 mg/kg biweekly in patients with mild to moderate AD reduced brain amyloid levels. The treatment also significantly slowed cognitive decline, as assessed by ADCOMS, CDR-SB, and ADAS-Cog, with the treatment group achieving lower scores on all three scales. The differences were statistically significant. Similarly, clinical studies have shown that lecanemab significantly reduces amyloid burden on PET. Notably, this reduction is associated with a slowing of cognitive decline in treated patients.

In this study, a total of 4 articles were included, and the mean change from baseline to post-treatment in ADCOMS was statistically analyzed in AD patients receiving lecanemab at 10 mg/kg biweekly, with the corresponding forest plot presented in Figure 2A. The assessment of heterogeneity among the included studies showed I^2^ = 0%, indicating no significant heterogeneity between the study results. Therefore, a fixed-effect model was used for the meta-analysis. The results showed that compared with the control group, the lecanemab treatment group had a significant decrease in ADCOMS scores (SMD = −0.1999, 95% CI [−0.2831, −0.1167], I^2^ = 0%, *P* < 0.00001), indicating that lecanemab significantly improves the composite cognitive function score in patients with AD.

**FIGURE 2:**
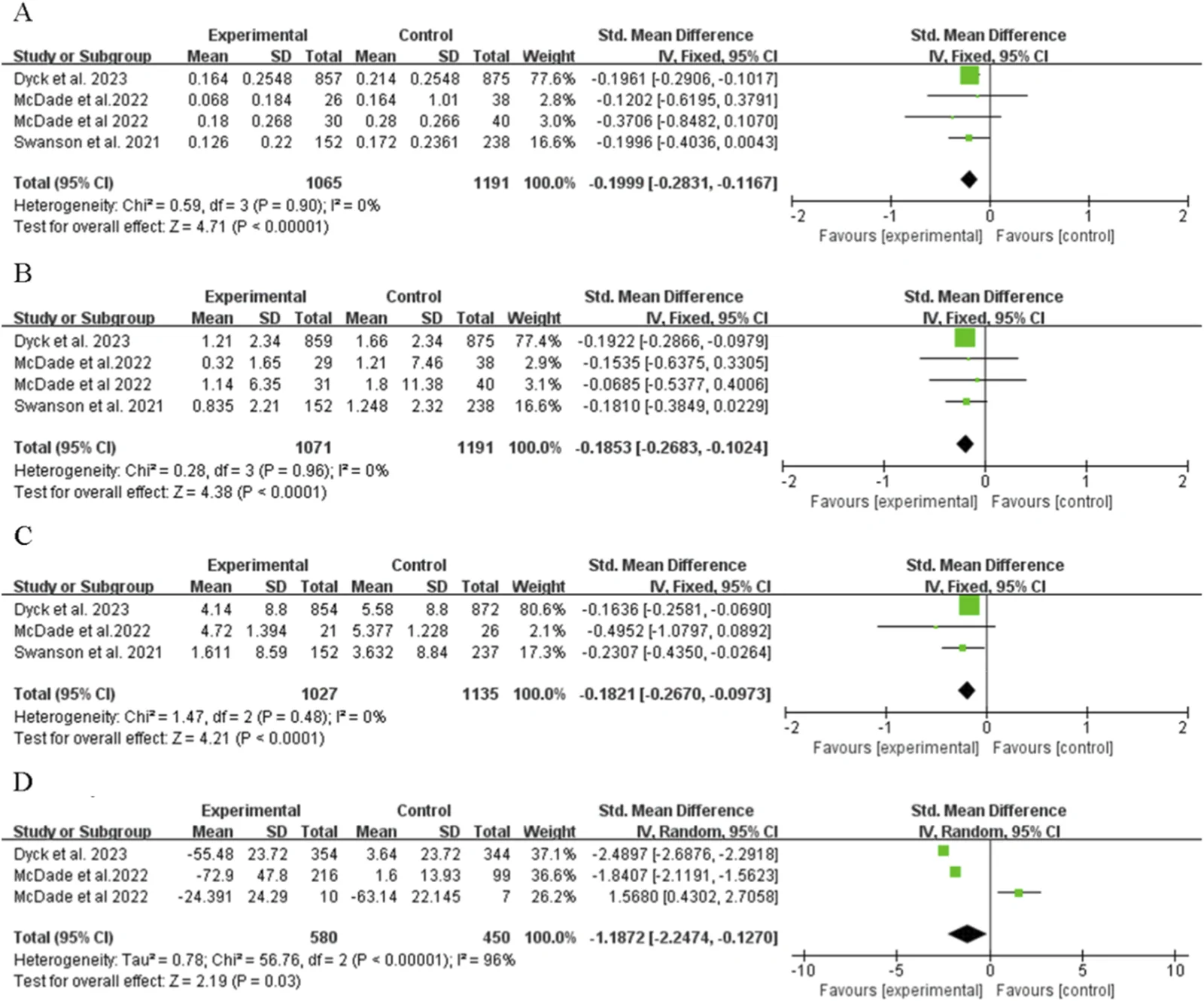
Meta-analysis of risk differences in efficacy outcomes between lecanemab and control groups. **(A)** ADCOMS **(B)** CDR-SB **(C)** ADAS-Cog **(D)** Amyloid burden on PET.

In this study, a total of 4 articles were included, and the mean change from baseline to post-treatment in CDR-SB was statistically analyzed in AD patients receiving lecanemab at 10 mg/kg biweekly, with the corresponding forest plot presented in Figure 2B. The assessment of heterogeneity among the included studies showed I^2^ = 0%, indicating no significant heterogeneity between the study results. Therefore, a fixed-effect model was used for the meta-analysis. The results showed that compared with the control group, the lecanemab treatment group had a significant decrease in CDR-SB scores (SMD = −0.1853, 95% CI [-0.2683, −0.1024], I^2^ = 0%, *P* < 0.0001), indicating that lecanemab significantly improves the severity of dementia in patients with AD compared with the control group.

In this study, a total of 3 RCTs were included, and the mean change from baseline to post-treatment in ADAS-Cog was statistically analyzed in AD patients receiving lecanemab at 10 mg/kg biweekly, with the corresponding forest plot presented in Figure 2C. The assessment of heterogeneity among the included studies showed I^2^ = 0%, indicating no significant heterogeneity between the study results. Therefore, a fixed-effect model was used for the meta-analysis. The results showed that compared with the control group, the lecanemab treatment group had a significant decrease in ADAS-Cog scores (SMD = −0.1821, 95% CI [-0.2670, −0.0973], I^2^ = 0%, *P* < 0.0001), indicating that lecanemab treatment leads to significant improvement or slowing of cognitive decline in patients with AD compared with the control group.

In this study, a total of 3 RCTs were included, and the mean change from baseline to post-treatment in amyloid burden on PET was statistically analyzed in AD patients receiving lecanemab at 10 mg/kg biweekly, with the corresponding forest plot presented in Figure 2D. The assessment of heterogeneity among the included studies showed I^2^ = 96%, indicating significant heterogeneity between the study results. Therefore, a random-effect model was used for the meta-analysis. The results showed that compared with the control group, the lecanemab treatment group had a significant reduction in amyloid burden on PET scores (SMD = −1.1872, 95% CI [-2.2474, −0.1270], I^2^ = 96%, *P* = 0.03), indicating that lecanemab significantly intervenes in the pathological progression of AD. Sensitivity analysis using the fixed-effect model yielded an SMD of −2.1962 (95% CI [−2.3559, −2.0365], I^2^ = 96%, *P* < 0.00001), which was directionally consistent with the random-effects estimate but more extreme in magnitude, which was directionally consistent with the random-effects estimate but more extreme in magnitude. Given the substantial heterogeneity (I^2^ = 96%), the random-effects model result was considered the primary finding, and the fixed-effect model result should be interpreted with caution. In addition, it is noteworthy that one subgroup from the McDade et al. (2022) study showed an atypical positive effect, which was derived from a small exploratory subgroup of ApoE ε4 homozygotes (n = 10 in the treatment arm and n = 7 in the placebo arm) who received 10 mg/kg biweekly lecanemab. Due to a protocol amendment during the trial, enrollment of this subgroup was limited, and the mean treatment duration was only approximately 173 days. The substantial amyloid reduction observed in the control arm is most likely attributable to the extremely small sample size, baseline differences, and/or regression to the mean, rather than a true treatment effect. Therefore, this result should be interpreted with caution.

In summary, compared with the control group, treatment with lecanemab in patients with AD resulted in lower scores on ADCOMS, CDR-SB, ADAS-Cog, and amyloid burden on PET. These reductions were associated with a slowing of the progression of dementia severity. Except for amyloid burden on PET, no significant asymmetry was observed for the other outcomes, indicating no substantial publication bias. The corresponding funnel plots are presented in Supplementary Figure S2.

### 3.3 Meta-analysis results of the safety (secondary outcomes) of lecanemab in patients with mild to moderate AD

Regarding safety outcomes, a total of five trials provided statistical data on the risk of ARIA-E as an adverse event associated with lecanemab treatment at 10 mg/kg biweekly in AD patients. Among 1,251 patients in the experimental group, 143 reported ARIA-E as an adverse event, while among 1,211 patients in the control group, 21 reported ARIA-E as an adverse event. Based on the fixed-effect model, a meta-analysis of the five included studies was performed, and the forest plot is presented in Figure 3A. The results showed that compared with the control group, lecanemab treatment for AD was associated with a significantly increased overall risk of ARIA-E as an adverse event (MH RR 5.9948, 95% CI [3.9047, 9.2037], I^2^ = 86%, *P* < 0.00001). The same five trials also reported the risk of ARIA-H as an adverse event. Among 1,116 patients in the experimental group, 171 reported ARIA-H as an adverse event, while among 1,346 patients in the control group, 119 reported ARIA-H as an adverse event. Based on the fixed-effect model, a meta-analysis of the five included studies was performed, and the forest plot is presented in Figure 3B. The results showed that compared with the control group, lecanemab treatment at 10 mg/kg biweekly for AD was associated with an increased overall risk of ARIA-H as an adverse event (MH RR 1.7490, 95% CI [1.3896, 2.2014], I^2^ = 41%, *P* < 0.00001). In addition, 2 RCTs provided statistical data on the risk of death associated with lecanemab treatment at 10 mg/kg biweekly in AD patients. Among 1,059 patients in the experimental group, 6 deaths were reported, while among 1,142 patients in the control group, 9 deaths were reported. Based on the fixed-effect model, a meta-analysis of the 2 included RCTs was performed, and the forest plot is presented in Figure 3C. The results showed that compared with the control group, lecanemab treatment for AD was not associated with a significantly increased overall risk of death as an adverse event (MH RR 0.7342, 95% CI [0.2673, 2.0166], I^2^ = 0%, *P* = 0.55).

**FIGURE 3:**
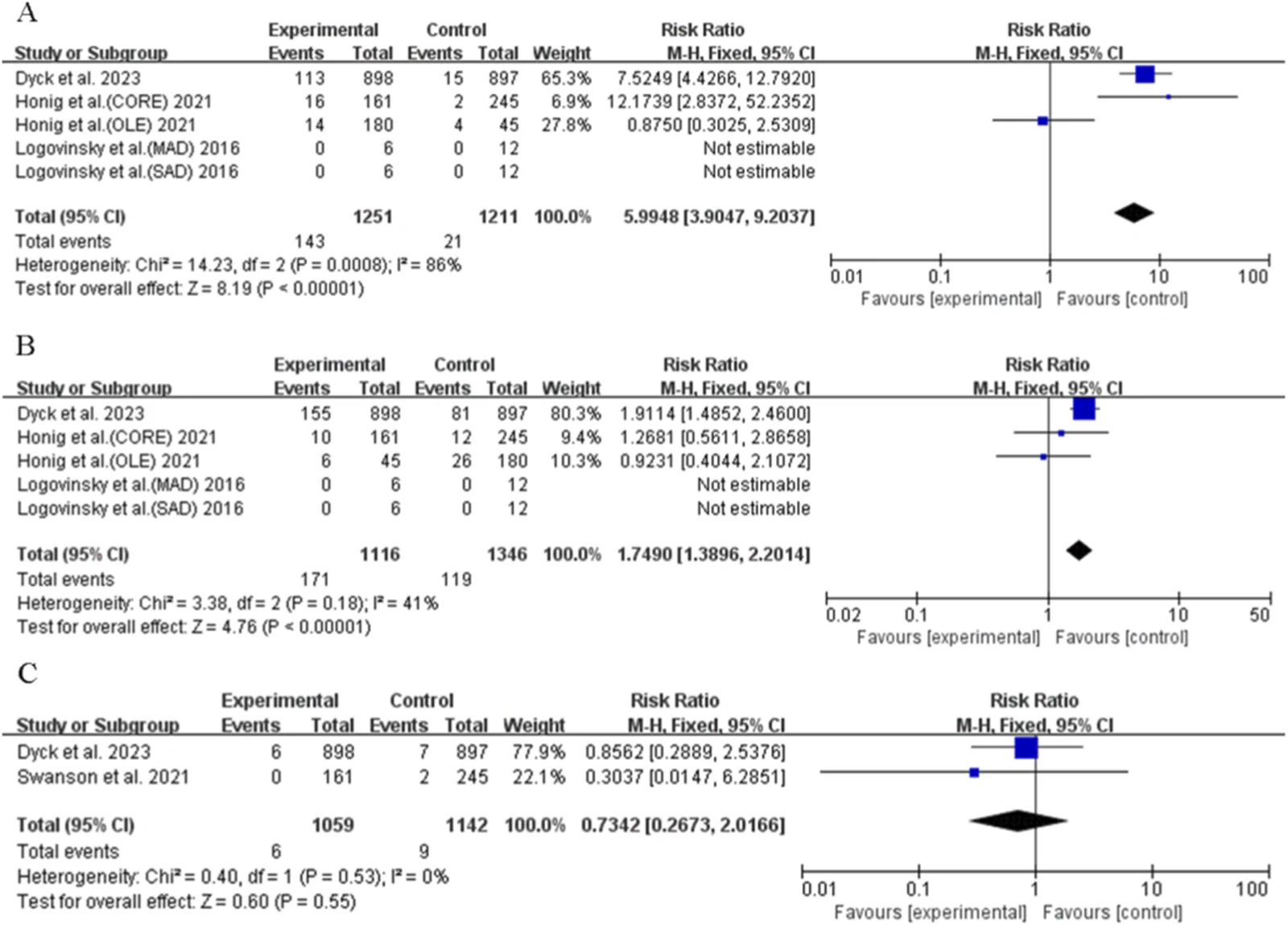
Meta-analysis of risk differences in safety outcomes between lecanemab and control groups. **(A)** ARIA-E **(B)** ARIA-H **(C)** Death.

In terms of symptom-related safety, we performed separate meta-analyses based on a fixed-effects model for safety outcomes reported across different RCTs, including TEAEs, headache, dizziness, and urinary tract infections.

A total of 4 RCTs reported data on the risk of TEAEs in patients with AD receiving lecanemab at 10 mg/kg biweekly. Relevant data were extracted, and a meta-analysis was conducted using a fixed-effects model across the 4 included studies. The corresponding forest plot is presented in Figure 4A. The results showed that, compared with the control group, lecanemab was associated with a significantly increased overall risk of TEAEs, with low heterogeneity (MH RR 1.0730, 95% CI [1.0296, 1.1183], I^2^ = 0%, *P* = 0.0008), suggesting a potential risk of adverse events during treatment. A total of 2 RCTs reported data on the risk of headache as an adverse event in patients with AD receiving lecanemab at 10 mg/kg biweekly, and the relevant data were extracted. A meta-analysis was conducted using a fixed-effects model based on the 2 included studies, and the corresponding forest plot is presented in Figure 4B. The results indicated that, compared with the control group, lecanemab at 10 mg/kg biweekly was associated with a significantly increased risk of headache, with low heterogeneity (MH RR 1.3617, 95% CI [1.0243, 1.8103], I^2^ = 0%, *P* = 0.03), suggesting a potential risk of headache during treatment.

**FIGURE 4:**
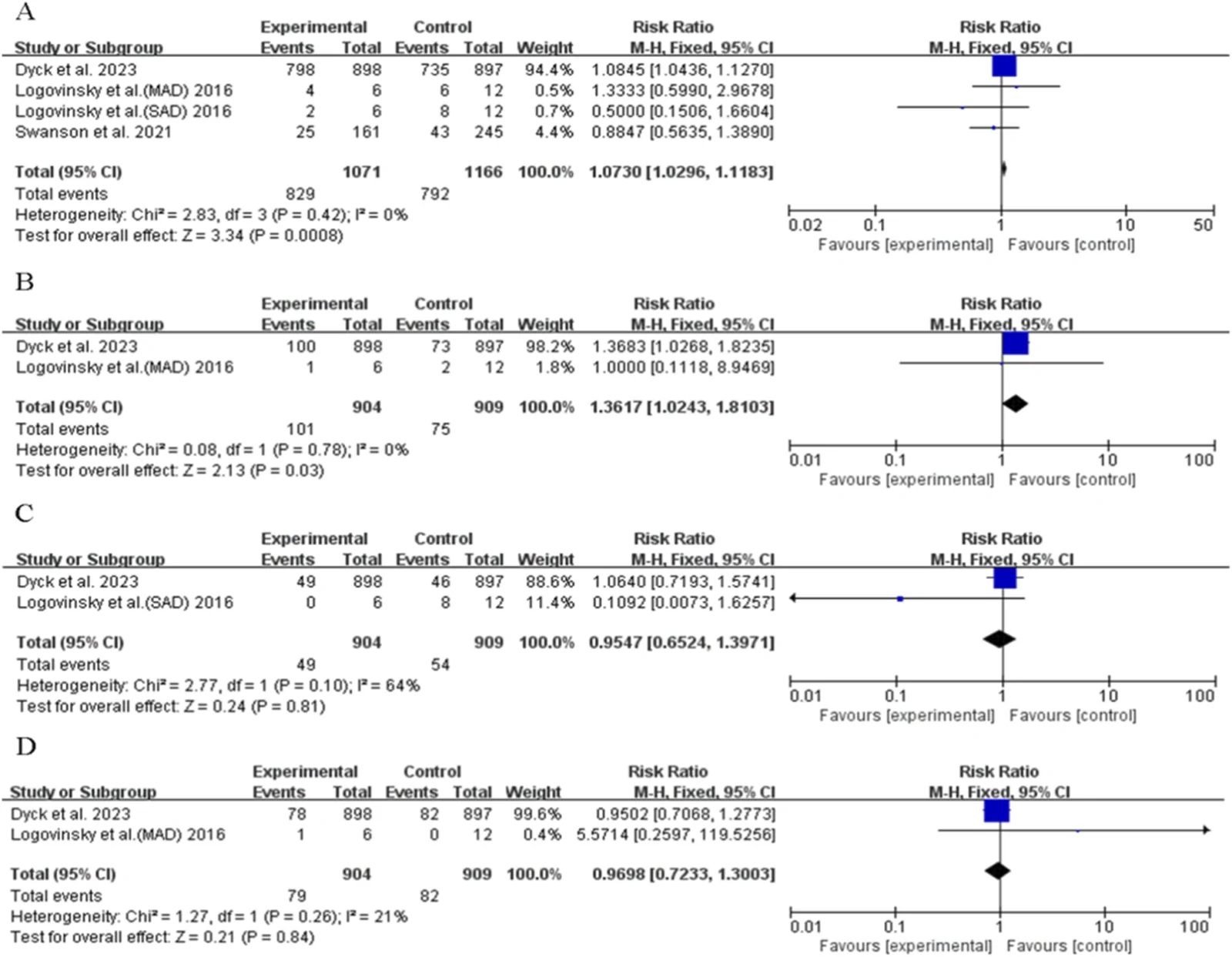
Meta-analysis of risk differences in symptom-related safety outcomes between lecanemab and control groups. **(A)** TEAE **(B)** Headache **(C)** Dizziness **(D)** Urinary tract infection.

A total of 2 RCTs reported data on the risk of dizziness as an adverse event in patients with AD receiving lecanemab at 10 mg/kg biweekly, and the relevant data were extracted. A meta-analysis was conducted using a fixed-effects model based on the 2 included studies, and the corresponding forest plot is presented in Figure 4C. The results showed that, compared with the control group, lecanemab was not associated with a significant increase in the risk of dizziness (MH RR 0.9547, 95% CI [0.6524, 1.3971], I^2^ = 64%, *P* = 0.81). A total of 2 RCTs reported data on the risk of urinary tract infection as an adverse event in patients with AD receiving lecanemab at 10 mg/kg biweekly, and the relevant data were extracted. A meta-analysis was conducted using a fixed-effects model based on the 2 included studies, and the corresponding forest plot is presented in Figure 4D. The results showed that, compared with the control group, lecanemab was not associated with a significant increase in the risk of urinary tract infection (MH RR 0.9698, 95% CI [0.7233, 1.3003], I^2^ = 21%, *P* = 0.84). These findings suggest that, relative to the control group, lecanemab treatment did not show significant differences in the risks of dizziness or urinary tract infection.

In summary, regarding safety outcomes, compared with the control group, patients with AD receiving lecanemab at 10 mg/kg biweekly showed significant differences in the incidence of ARIA-E and ARIA-H, although a certain degree of heterogeneity was observed. In contrast, no significant difference was found in death, a serious safety outcome, and no heterogeneity was detected. The funnel plots for ARIA-E, ARIA-H, and death showed no substantial asymmetry, indicating no apparent publication bias, as presented in Supplementary Figure S3. Regarding symptom-related safety outcomes, compared with the control group, patients with AD receiving lecanemab at 10 mg/kg biweekly showed a significantly increased risk of overall TEAEs and headache. In contrast, no significant differences were observed in the risks of dizziness or urinary tract infection. In addition, due to the limited number of studies, funnel plot analysis could not be performed for symptom-related safety outcomes. Therefore, no funnel plots or publication bias assessments were conducted for this part of the analysis to avoid unreliable conclusions resulting from an insufficient number of studies.

### 3.4 Sensitivity analyses

We conducted a total of 11 sensitivity analyses across 4 efficacy-related cognitive outcome measures and 7 safety-related indicators, and the corresponding results are presented in Supplementary Figures S4-S6.

#### 3.4.1 Sensitivity analysis of efficacy outcomes (primary outcomes)

A meta-analysis was performed on the 4 included clinical trials, with pooled analyses conducted using both fixed-effects and random-effects models. The results showed that, for the 2 cognitive outcome measures ADCOMS and CDR-SB, the pooled effect estimates were identical between the fixed-effects and random-effects models (ADCOMS SMD = −0.1999, 95% CI [-0.2831, −0.1167], I^2^ = 0%, *P* < 0.00001; CDR-SB SMD = −0.1853, 95% CI [-0.2683, −0.1024], I^2^ = 0%, *P* < 0.0001). A meta-analysis was also conducted on the 3 included RCTs using both fixed-effects and random-effects models for pooling. The results showed that, for the ADAS-Cog cognitive outcome measure, the pooled effect estimates were identical between the fixed-effects and random-effects models (ADAS-Cog SMD = −0.1821, 95% CI [-0.2670, −0.0973], I^2^ = 0%, *P* < 0.0001). These findings indicate extremely low heterogeneity among the 3 outcomes, suggesting that the choice of statistical model has no substantive impact on the results. To further assess the stability of these findings, sensitivity analyses were performed by excluding the two phases from the Livingston et al. (2024) study to evaluate whether treating these phases as independent RCTs influenced the overall estimates. The results showed that the direction and significance of the pooled effect estimates for all cognitive outcomes remained unchanged after their exclusion (ADCOMS SMD = −0.1999, 95% CI [-0.2831, −0.1167], I^2^ = 0%, *P* < 0.00001; CDR-SB SMD = −0.1853, 95% CI [-0.2683, −0.1024], I^2^ = 0%, *P* < 0.0001), confirming that our primary findings were not unduly influenced by the inclusion of these split-phase comparisons. In contrast, the pooled results for amyloid burden on PET exhibited substantial heterogeneity (I^2^ = 96%,τ^2^ = 0.78, *P* < 0.00001), with notable differences in effect estimates between the fixed-effects and random-effects models (fixed-effects model: SMD = −2.1962, 95% CI [-2.3559, −2.0365], *P* < 0.00001). To further assess the stability of these findings, sensitivity analyses were performed. After sequentially removing individual studies, the effect sizes for ADCOMS, CDR-SB, and ADAS-Cog remained stable (SMD ranging from −0.20 to −0.18, with largely overlapping 95%CIs and I^2^ consistently at 0%), indicating consistent results. In contrast, in the analysis of amyloid burden on PET, exclusion of any individual small-sample study had a noticeable impact on the pooled effect. In particular, McDade et al. (McDade et al., 2022) contributed substantially to the overall results. This study had a relatively large sample size and showed marked differences in PET changes in the treatment group, thereby exerting a dominant influence on the overall pooled effect estimate.

#### 3.4.2 Sensitivity analysis of safety outcomes (secondary outcomes)

A pooled analysis of the five included clinical trials was conducted using both fixed-effect and random-effect models. The results showed that the pooled estimates for ARIA-E and ARIA-H had poor stability, whereas the outcome of death was relatively stable. Under the fixed-effect model, the RR for ARIA-E was 5.9948 (95% CI [3.9047, 9.2037], *P* < 0.00001), and the RR for ARIA-H was 1.7490 (95% CI [1.3896, 2.2014], *P* < 0.00001). However, both outcomes exhibited high heterogeneity (I^2^ = 86% and 41%, respectively). When the random-effect model was applied, the effect size estimates changed substantially and were no longer statistically significant for either outcome (ARIA-E: RR = 4.2367, 95% CI [0.9378, 19.1393], *P* = 0.06; ARIA-H: RR = 1.5136, 95% CI [0.9762, 2.3468], *P* = 0.06). Further analysis using the leave-one-out method revealed that the heterogeneity was primarily attributable to the Honig (OLE) study (Honig et al., 2023), whose open-label extension design led to effect estimates inconsistent with those of the other RCTs. A pooled analysis of the 2 RCTs reporting death as an outcome was conducted using both fixed-effect and random-effect models. The results indicated that the pooled estimate for death was relatively stable. Under the fixed-effect model, the RR for death was 0.7342 (95% CI [0.2673, 2.0166], *P =* 0.55); under the random-effect model, the RR was 0.7608 (95% CI [0.2736, 2.1157], *P* = 0.06). Both models showed no statistically significant difference in risk between groups, with very low heterogeneity (I^2^ = 0%).

For the TEAE outcome, the 4 included RCTs were pooled using both fixed-effects and random-effects models. The results demonstrated that the pooled estimates for TEAE were stable (fixed-effects model: RR = 1.0730, 95% CI [1.0296, 1.1183], *P* = 0.0008; random-effects model: RR = 1.0826, 95% CI [1.0419, 1.1248], *P* < 0.0001). Both models indicated no statistically significant difference in risk between groups, and the results showed extremely low heterogeneity (I^2^ = 0%). A pooled analysis of the 2 RCTs reporting headache as an outcome was conducted using both fixed-effect and random-effect models. The results indicated that the pooled estimate for headache was relatively consistent. Under the fixed-effect model, the RR was 1.3617 (95% CI [1.0243, 1.8103], *P* = 0.03); under the random-effect model, the RR was 1.3611 (95% CI [1.0238, 1.8095], *P* = 0.03). Heterogeneity was very low (I^2^ = 0%), indicating that both results were stable and reliable, and the incidence of headache was significantly higher in the experimental group than in the control group. For the dizziness and urinary tract infection outcomes, the pooled results from the 2 included RCTs showed no statistically significant differences under either model.

## 4 Discussion

AD is the most common age-related neurodegenerative disorder and is characterized by distinct neuropathological features in the brain, including extracellular plaques composed of amyloid-β (Aβ) and intracellular neurofibrillary tangles formed by tau protein (Ono and Tsuji, 2020; Niedowicz et al., 2011). Aβ exists in multiple forms, including monomers, oligomers, protofibrils, and insoluble fibrils within plaques. Oligomers and protofibrils are neurotoxic, and the removal of these aggregates may represent an effective therapeutic strategy for AD (Söderberg et al., 2023). Lecanemab is a humanized IgG1 monoclonal antibody. Reducing Aβ deposition has been demonstrated to be an effective strategy for slowing cognitive decline in AD. Its unique pharmacological advantage lies in its high-affinity and selective binding to Aβ protofibrils, enabling efficient targeting and clearance of these highly toxic aggregates (Kim et al., 2025; Johannesson et al., 2024).

The results of this meta-analysis are consistent with the aforementioned mechanism of action of lecanemab and its positive findings across multiple clinical trials. Based on a fixed-effects model, the analysis showed that, compared with the control group, patients with AD receiving lecanemab achieved significant improvements across cognitive and functional assessment scales, including ADCOMS (SMD = −0.1999), CDR-SB (SMD = −0.1853), and ADAS-Cog (SMD = −0.1821), with all *P* values <0.0001. These findings indicate that lecanemab can slow cognitive decline and reduce dementia severity in patients with AD. Such results are supported by multiple independent studies. For example, in the pivotal Clarity AD phase III trial, lecanemab demonstrated significant clinical benefit on the primary endpoint of CDR-SB, reducing cognitive decline by 27% over an 18-month period (van D et al., 2023). Similarly, a systematic review and meta-analysis of lecanemab also reported its positive effects on clinical outcomes (Abdelazim et al., 2024). Notably, the present study further confirms the direct impact of lecanemab on the pathological progression of AD. The meta-analysis in this study showed that the lecanemab treatment group exhibited a significant reduction in amyloid burden on PET (SMD = −1.1872, *P* = 0.03). Although substantial heterogeneity was observed for this outcome (I^2^ = 96%), the core findings are consistent with the phase II study reported by McDade et al. (2022), which demonstrated dose-dependent and time-dependent reductions in cerebral amyloid following lecanemab treatment, along with corresponding clinical benefits. This further supports the Aβ protofibril hypothesis, suggesting that the clearance of pathological amyloid in the brain can effectively slow the clinical progression of AD. In addition, a recently published single-arm real-world study in the Chinese AD population Liao et al. (Liao et al., 2026) reported that reductions in blood-based biomarkers were significantly correlated with Aβ-PET clearance, with findings directionally consistent with the results of our meta-analysis, further reinforcing the consistency of our conclusions.

Although our meta-analysis demonstrated statistically significant differences in the primary cognitive outcomes favoring lecanemab over placebo, the clinical meaningfulness of these differences warrants careful interpretation. The concept of minimal clinically important difference (MCID) provides a framework to distinguish statistical significance from clinically meaningful benefit. For the CDR-SB, the MCID has been estimated to range from approximately 1–2 points over 18 months (Muir et al., 2024). In the pivotal CLARITY-AD trial (van D et al., 2023), the mean difference in CDR-SB between lecanemab and placebo was −0.45 (95% CI: −0.67 to −0.23), which is below this MCID threshold. Our pooled estimates, which are consistent with these trial-level findings, suggest that the magnitude of cognitive benefit associated with lecanemab, while statistically significant, may not reach the threshold generally considered clinically important in the overall population.

This observation aligns with findings from a recent systematic review and meta-analysis of donanemab by Hu and Zhang, which reported that although donanemab significantly reduced cognitive decline, with a mean difference of +2.93 points on the iADRS and −0.66 points on the CDR-SB, these improvements did not approach their respective MCID thresholds (Hu and Zhang, 2026). Collectively, these data underscore the need for caution when interpreting statistically significant results as clinically meaningful. Future research should prioritize the establishment of validated MCID thresholds specific to early AD populations and should incorporate patient-centered outcomes to better capture the real-world relevance of treatment effects. Nevertheless, the clinical significance of lecanemab should also be considered in the context of its sustained effect over time and the potential for cumulative benefit with longer treatment duration.

Drug safety is a key factor in evaluating its clinical value. This meta-analysis comprehensively assessed the safety profile of lecanemab, with particular attention to ARIA, TEAEs, and headache. For death, dizziness, and urinary tract infection, the analyses were based on a limited number of studies, and the findings should be interpreted as inconclusive rather than as evidence of safety. The analysis showed that lecanemab treatment significantly increased the risk of ARIA-E (MH RR = 5.9948, *P* < 0.00001) and ARIA-H (MH RR = 1.7490, *P* < 0.00001). These findings are consistent with previously published studies, such as the work by Honig et al. (2023), which specifically investigated the safety of ARIA associated with lecanemab and provided detailed evidence on its incidence in phase II trials and open-label extension studies, as well as related risk factors, including the APOE ε4 genotype, and strategies for clinical management.

However, the sensitivity analysis of ARIA outcomes in this study revealed issues with the stability of the results. We found significant heterogeneity in the meta-analyses of both ARIA-E and ARIA-H (I^2^ = 86% and 41%, respectively). After sequential exclusion, the heterogeneity was primarily attributed to the open-label extension study reported by Honig et al. (Honig et al., 2023). This study differed substantially in design from the other included RCTs, potentially introducing time bias and selection bias. This heterogeneity suggests that the results of the fixed-effect model may have been overestimated. When a more conservative random-effect model was applied, the risk associations for both ARIA-E and ARIA-H were no longer statistically significant (ARIA-E: *P* = 0.06; ARIA-H: *P* = 0.06), indicating that greater caution is warranted when interpreting results from meta-analyses that include heterogeneous studies.

In addition to ARIA, this study also found that lecanemab treatment was associated with an increased risk of overall TEAEs (MH RR = 1.0730, *P* = 0.0008) and headache (MH RR = 1.3617, *P* = 0.03). These findings had very low heterogeneity (I^2^ = 0%), indicating that the results are stable and reliable. Headache, as a common adverse reaction, may be associated with perivascular inflammatory responses related to lecanemab treatment. Furthermore, based on a fixed-effects model, this study found no significant association between lecanemab treatment and the risks of death (MH RR = 0.7342, *P* = 0.55), dizziness (MH RR = 0.9547, *P* = 0.81), or urinary tract infection (MH RR = 0.9698, *P* = 0.84). These results were further confirmed by the random-effects model, indicating consistency of the findings. For death, dizziness, and urinary tract infection, the analyses were based on a limited number of studies (only two RCTs each), and the non-significant findings should be interpreted as inconclusive rather than as evidence of safety.

This study provides a comprehensive meta-analysis of the efficacy and safety data of lecanemab in the treatment of mild to moderate AD, and the consistency of the results was further confirmed through rigorous sensitivity analyses. Regarding the heterogeneity observed in the meta-analysis, we identified its primary source using the leave-one-out method and discussed the impact of different statistical models on the results, providing useful methodological insights for future research. However, this study has several limitations. First, the RCTs included in this meta-analysis enrolled patients with mild to moderate AD, whereas the approved indication for lecanemab is specifically limited to MCI and mild AD. Therefore, the inclusion of moderate AD patients may limit the generalizability of our findings to the approved target population, and our results should be interpreted with caution when applied to the MCI and mild AD population alone. Second, for some safety outcomes, such as dizziness and urinary tract infection, the limited number of included studies precluded reliable publication bias testing, and the results should be interpreted with caution. Third, although we conducted a detailed analysis of the sources of heterogeneity for ARIA outcomes, we were unable to fully eliminate their influence due to inherent limitations in the available data. In addition, it should be noted that the CORE data from the phase 2 study by McDade et al. (2022) and the OLE data reported by Honig et al. (2023) are derived from the same patient cohort. While we included OLE data in the safety analysis to maximize the available information on rare events such as ARIA, these data lack a concurrent control group and are subject to inherent biases associated with open-label extensions. Therefore, the OLE-derived safety results should be interpreted with caution, and the primary conclusions regarding safety are based on the RCT data. This approach is consistent with the methodological framework of a previously published systematic review and meta-analysis of lecanemab, which similarly included OLE data alongside RCTs to maximize safety information (Arroyo-Pacheco et al., 2025). Additionally, the split phases from the Logovinsky et al. (Livingston et al., 2024) study were treated as independent RCTs in our primary analysis. Although this approach was based on the original study design, which reported each phase as a separate randomized comparison with distinct participant cohorts, and sensitivity analyses excluding these phases confirmed the stability of our findings, we acknowledge that this may have introduced some degree of statistical dependence. Therefore, the results should be interpreted with this consideration in mind. Furthermore, due to the limited availability of outcome data stratified by APOE ε4 carrier status in the included studies, we were unable to perform a formal subgroup meta-analysis to examine whether the efficacy and safety of lecanemab differ between carriers and non-carriers, which we acknowledge as another limitation of the current meta-analysis. Future studies with standardized reporting of APOE ε4-stratified outcomes and more detailed subgroup analyses incorporating key risk factors are warranted. Future studies may also consider analyzing trials with different study designs separately in subgroup analyses to obtain more homogeneous pooled effect estimates.

## Data Availability

The original contributions presented in the study are included in the article/Supplementary Material, further inquiries can be directed to the corresponding authors.

## Supplementary material

SUPPLEMENTARY TABLE S1 Search strategy: Taking PubMed as an example.

SUPPLEMENTARY FIGURE S1 Risk of bias assessment of studies.

SUPPLEMENTARY FIGURE S2 Funnel plots of efficacy outcomes for lecanemab in patients with AD.

SUPPLEMENTARY FIGURE S3 Funnel plots of safety outcomes for lecanemab in patients with AD.

SUPPLEMENTARY FIGURE S4 Sensitivity analysis of the efficacy of lecanemab in patients with AD based on different statistical models.

SUPPLEMENTARY FIGURE S5 Sensitivity analysis of the safety of lecanemab in patients with AD based on different statistical models.

SUPPLEMENTARY FIGURE S6 Sensitivity analysis of symptom-related safety outcomes of lecanemab in patients with AD based on different statistical models.
