# Supplementary material for: Efficacy and safety of lecanemab monoclonal antibody in the treatment of mild to moderate Alzheimer’s disease: a systematic review and meta-analysis
Source: Front Pharmacol. 2026 Sep 28;17:1947129. doi: 10.3389/fphar.2026.1947129 (PMC13655067; doi:10.3389/fphar.2026.1947129)
Supplement: Supplementary file 1 [file Table1.docx]

**Supplemental Table 1. Search strategy:** Taking PubMed as an example

("Alzheimer Disease"[Mesh] OR "Alzheimer Disease" OR "Alzheimer Syndrome" OR "Alzheimer-Type Dementia" OR "ATD" OR "Alzheimer's Disease" OR "Alzheimer Dementia" OR "Senile Dementia" OR "Alzheimer Type Dementia" OR "Primary Senile Degenerative Dementia" OR "Alzheimer Sclerosis" OR "Sclerosis, Alzheimer" OR "Presenile Dementia" OR "Acute Confusional Senile Dementia" OR "Familial Alzheimer Disease" OR "FAD") AND (lecanemab OR leqembi OR BAN2401 OR BAN 2401 OR BAN-2401)
