# Supplementary material for: Efficacy and safety of lecanemab monoclonal antibody in the treatment of mild to moderate Alzheimer’s disease: a systematic review and meta-analysis
Source: Front Pharmacol. 2026 Sep 28;17:1947129. doi: 10.3389/fphar.2026.1947129 (PMC13655067; doi:10.3389/fphar.2026.1947129)
Supplement: Supplementary file 2 [file Supplementaryfile1.doc]

Supplement figure

Figure S1 Risk of bias assessment of studies

Figure S2 Funnel plots of efficacy outcomes for lecanemab in patients with AD

(a)ADCOMS (b)CDR-SB (c)ADAS-Cog (d)Amyloid burden on PET

Figure S3 Funnel plots of safety outcomes for lecanemab in patients with AD

(a)ARIA-E (b)ARIA-H (c)Death

Figure S4 Sensitivity analysis of the efficacy of lecanemab in patients with AD based on different statistical models

(A) ADCOMS (B) CDR-SB (C)ADAS-Cog (D)Amyloid burden on PET

Figure S5 Sensitivity analysis of the safety of lecanemab in patients with AD based on different statistical models

(A)ARIA-E (B)ARIA-H (C)Death

Figure S6 Sensitivity analysis of symptom-related safety outcomes of lecanemab in patients with AD based on different statistical models

(A)TEAE (B)Headache (C)Dizziness (D)Urinary tract infection

Figure S1 Risk of bias assessment of studies

Figure S2 Funnel plots of efficacy outcomes for lecanemab in patients with AD

(a)ADCOMS (b)CDR-SB (c)ADAS-Cog (d)Amyloid burden on PET

Figure S3 Funnel plots of safety outcomes for lecanemab in patients with AD

(a)ARIA-E (b)ARIA-H (c)Death

Figure S4 Sensitivity analysis of the efficacy of lecanemab in patients with AD based on different statistical models

(A) ADCOMS (B) CDR-SB (C)ADAS-Cog (D)Amyloid burden on PET

Figure S5 Sensitivity analysis of the safety of lecanemab in patients with AD based on different statistical models

(A)ARIA-E (B)ARIA-H (C)Death

Figure S6 Sensitivity analysis of symptom-related safety outcomes of lecanemab in patients with AD based on different statistical models

(A)TEAE (B)Headache (C)Dizziness (D)Urinary tract infection
